# 

**Published:** 1890-03-15

**Authors:** 


SATURDAY, MARCH 15th, 1890.

**An Impeached Committee** - 367
**Words of Consolation.**—Perseverance - 368
**The Town Dweller.**—Life in Town and Country - 369
**The Hospital Problem.** By MR. HENRY C. BURDETT. II.—What is Wanted - 370
**The Doctor's Pulpit.**—Unstable Mental Equilibrium - 371
**The Right to Perform Operations in Hospitals** - 372
**Everybody's Page** - 373
**Notes and News** - 374
**Hospital Book-keeping and Accounts** - 375
**Annotations.**—A New Danger from Tobacco—A New Pain Killer—The Princess of Wales and Nurses' Pensions - 376
**Doctors' Fees in Germany** - 380
**The Practitioner's Mirror and Retrospect:**
MEDICINE—Herpes Zoster—Acne—Dentition—Temperature 377
SURGERY—Injuries of the Neck of the Femur—Fracture of the Patella — Vaccination — Hernia — Mikulicz's Operation - 378
THERAPEUTICS—Electricity - 380
NEW DRUGS, APPLIANCES, AND THINGS MEDICAL - 380
**Statistics of Insanity in England** - 381
**Turning Over New Leaves**—James Vraille - 381
**Editor's Letter Box**—Ought Members of the Active Medical Staff to Sit upon Hospital Boards? - 381
**Passing Topics.**—"Working Men" and Hospital Committees 382
**Scraps and Gleanings** - 382

### EXTRA SUPPLEMENT—THE NURSING MIRROR.

**En Passant.**—Jersey Institution—Honours for the First Thousand—Popular Nursing—Thousands of Pounds for the National Pension Fund—Ely District Nursing—York Home for Nurses—A Matron's Grumble—The Scarborough Nurses' Institute xcvii

**The National Pension Fund for Nurses.**—A Donation Bonus of £40,000 - xcviii

**Between Cases** - xcix

**Everybody's Opinion.**—The Title of Nurse—Meum and Tuum - xcix

**Notes and Queries** - c

**Appointments** - c

**Vacancies** - c

**Amusements and Relaxation** - c

